# The Actinic Opacity of London Air

**Published:** 1898-08-06

**Authors:** 


### The Actinic Opacity of London Air.

There is an old advertisement, we really forget the particular patent starch, or what not, whose inventor first used it, but it runs to this effect: "When you ask for so-and-so, see that you get it!" This looks like common-sense, and it has far-reaching applications. When we go for our holidays and seek for health in sunshine we must remember that just as all is not gold that glitters, so all the sunshine which appears in the meteorological reports is not really sunshine in the full meaning of the term. There are various tepid modifications of sunshine which may serve well enough for statistical purposes, but, for all that, are very different from the glorious life-giving stream of energy which pours down upon those who visit the high Alps, or go yachting, or even visit the country in their own land. Several methods are in use by which the duration and intensity of sunshine are automatically recorded. But the duration of such sunlight as will make a mark is not of great importance, and its intensity, when measured by its heating and burning effect, is not of material moment. When sunshine tans the skin, when it takes photographs, when it fades carpets, when it destroys microbes, when it renders disease-germs sterile, it does so not by its duration or by its heat, but by virtue of its actinic power, and, unfortunately, this has not hitherto often been recorded.

Those who go about London getting paler month by month, and yet after a few days at the seaside find themselves tanned and blistered by the sun, must laugh at the published records according to which, however badly London comes out in the winter, the duration of bright sunshine during the summer months is not far short of that in the more favoured watering places. It is something in the quality of London sunshine which is lacking, so that we may say, in the words of the tradesmen, "When you ask for sunshine, see that you get it." Some years ago Dr. S. Rideal communicated to the British Association a very interesting paper on "The Iodine Value of Sunlight in the High Alps," in which he estimated the strength of sunlight not by its heating but by its actinic power, its capacity for inducing chemical change. No doubt, the heat of the sun is a great good, and without that supply of energy which is received from it in the form of sensible heat, life would be much harder; but what combines the elements, what falling upon the chlorophyll of vegetable life builds up the organic compounds upon which, ultimately, all other forms of life depend, is the actinic power which the sun's rays possess.

Recently Dr. Black Jones, of Llangammarch Wells, in Central Wales, has been making use of the same method for measuring the actinic power of the sun in that little watering place, and has been comparing it with what is found in London and in other places, and his results show how vastly superior country sunshine is in that most important particular to what is met with in towns. His experiments also tend to show that even in this country, so only that we go to places where the air is clear and transparent, we can find sunshine the power of which is as great as that which blisters the high Alps. At any rate, it was so in regard to Llangammarch, where the activity of the sun was found to be in no sense inferior to that which fell upon health resorts of very high altitude, such as St. Moritz. This matter is of some importance to those who are considering the choice of a health resort, but it is of still more moment to those whose fate it is to have to live in towns. Why is it that germs thrive in towns while they die out in the country? and why is it that the supply of ozone in our towns is always so deficient? The answer is that, however much the heat rays of the sun may permeate a smoky atmosphere, the actinic rays cannot get through.

When we go to the country, or the seaside, or to the high Alps for health, it is not more sunshine that we go in search of, for in summer London gets a fair supply; it is not more heat, for surely of heat London in August has enough. It is the full actinic power of clear sunshine that we seek—that actinism by the help of which the air should be kept pure and dust should be deprived of its morbific influence, but which, to our constant detriment, we filter out of London sunshine by the aid of smoke.

